# Metal Surface Treatments for Enhanced Heat Transfer in Metal–Composite Hybrid Structures

**DOI:** 10.3390/mi16040399

**Published:** 2025-03-29

**Authors:** Dong Hyun Kim, Wonhwa Lee, Jung Bin Park, Jea Uk Lee

**Affiliations:** Department of Advanced Materials Engineering for Information and Electronics, Integrated Education Institute for Frontier Science and Technology (BK21 Four), Kyung Hee University, Yongin-si 17104, Republic of Korea; spy04032@khu.ac.kr (D.H.K.); lwh980304@khu.ac.kr (W.L.); bin01@khu.ac.kr (J.B.P.)

**Keywords:** metal–polymer hybrid structures, heat dissipation, 3D printing, carbon composites, surface treatment techniques

## Abstract

Recently, there has been an increasing emphasis on improving the performance of metal components across various industries, such as automotive, aerospace, electronics, medical devices, and military applications. However, the challenges related to efficient heat generation and transfer in equipment and devices are becoming increasingly critical. A solution to these issues involves the adoption of a metal–composite hybrid structure, designed to efficiently manage heat, while substituting conventional metal components with polymer–carbon composites. In this study, nanopores were formed on the metal surface using an anodization process, serving as the basis for creating 3D-printed polymer/metal hybrid constructions. Various surface treatments, including plasma treatment, mixed electrolyte anodization, and etching, were applied to the metal surface to enhance the bonding strength between the 3D-printed polymer and the aluminum alloy. These processes were essential for developing lightweight polymer/metal hybrid structures utilizing a range of 3D-printed polymer filaments, such as polylactic acid, thermoplastic polyurethane, acrylonitrile butadiene styrene, polypropylene, thermoplastic polyester elastomer, and composite materials composed of polymer and carbon. In particular, the hybrid structures employing polymer–carbon composite materials demonstrated excellent heat dissipation characteristics, attributed to the remarkable conductive properties of carbon fibers. These technologies have the potential to effectively address the device heat problem by facilitating the development of lightweight hybrid structures applicable across various fields, including automotive, mobile electronics, medical devices, and military applications.

## 1. Introduction

The integration of metals and polymers through dissimilar joining has become a crucial area of research and development, propelled by the need for innovative material solutions across various industries. Metals are renowned for their exceptional strength and durability, while polymers are celebrated for their lightweight nature and resistance to corrosion. When these materials are combined, they complement each other, creating a synergy that is particularly advantageous in sectors such as automotives, aerospace, military, and electronics [1,2,3]. In these fields, the amalgamation of metals and polymers can lead to improved performance and the development of novel functionalities. Specifically, the joining of functional polymer composites with metals has opened up new possibilities for advanced applications. The distinctive properties of these hybrid structures, such as their improved mechanical strength, reduced weight, high electrical and thermal conductivity, and enhanced thermal stability, render them ideal for use in demanding environments [4,5]. The capacity to effectively join these materials not only expands their utility but also enables the design of complex structures that harness the advantages of both polymer composites and metals.

A crucial step in achieving strong dissimilar joining is the anodization of metal surfaces [6,7,8]. This electrochemical technique is essential for enhancing the adhesion between polymers and metals. Anodization involves creating a porous oxide layer on the metal surface, which facilitates mechanical interlocking with molten polymers during polymer molding processes, such as conventional injection molding and recently developed additive manufacturing methods (polymer 3D printing) [9,10,11]. In the metal anodization process, common electrolytes like sulfuric acid are used, which are known for producing small, well-ordered pores, and phosphoric acid, which generates larger pores suited for structural applications [12,13,14]. Additionally, oxalic acid is frequently employed for its ability to form medium-sized pores with a characteristic yellowish hue, contributing not only to the aesthetic appeal but also to the functional requirements of the joined structures [15].

To optimize the anodization process for combining metals and various polymer materials, it is essential to explore a wide range of electrolytes [16]. Each electrolyte contributes differently to the pore size and distribution, thereby affecting the bonding quality between the anodized metal surface and the polymer materials. For instance, the use of citric acid at high voltages can yield porous films with large cell diameters, while malonic acid is effective for creating larger cells at moderate voltages. Beyond anodization, post-treatment processes such as NaOH etching play a crucial role in modifying the metal surface [17]. This step enhances the surface morphology, increasing the overall adhesion strength between the metal and the polymer, which is vital for ensuring the longevity and reliability of the composite structures. In conclusion, the dissimilar joining of metals and polymers through anodization and subsequent treatments represent vital technological advancements. By carefully selecting and applying various electrolytes and post-treatment processes, it is possible to achieve highly durable and functional metal–polymer hybrid structures, poised to meet the evolving demands of modern engineering applications.

Very recently, our research group have explored a single-step anodizing treatment of aluminum using a mixed solution of phosphoric acid and hydrogen peroxide, followed by the direct injection molding and 3D printing of a polymer to achieve a strong metal–polymer hybrid structure [18]. The anodizing process has created a 3D channeling pore structure with uniform nanopores on the aluminum surface, significantly enhancing the bonding strength, which reached up to 40.34 MPa, among the highest reported for such joints [19]. This study has highlighted that hydrogen peroxide addition increases the nanopore size, facilitating better polymer (polylactic acid) penetration and interlocking. Moreover, the research has demonstrated an innovative application of 3D printing to join polylactic acid filament with anodized aluminum surfaces, eliminating the need for heavy molds and equipment, and achieving superior bonding through optimized nanopore sizes.

Our research group has reported significant advancements in enhancing the bonding strength of metal–polymer hybrid structures through the combined use of anodization and plasma treatment on aluminum surfaces [19,20]. The plasma treatment notably improved the surface chemistry by increasing the hydroxyl group content on the aluminum surface from 60.6% to 91.2%, thereby facilitating the better infiltration of polylactic acid during 3D printing on anodized surfaces. This improvement was supported by cross-sectional analysis via focused ion-beam etching, revealing the deeper polymer penetration into the treated aluminum layers. Consequently, the bonding strength between aluminum and 3D-printed polylactic acid was substantially enhanced, achieving 19.81 MPa after 10 min of plasma treatment, nearly doubling the 10.89 MPa observed in samples without plasma treatment.

**Figure 1:**
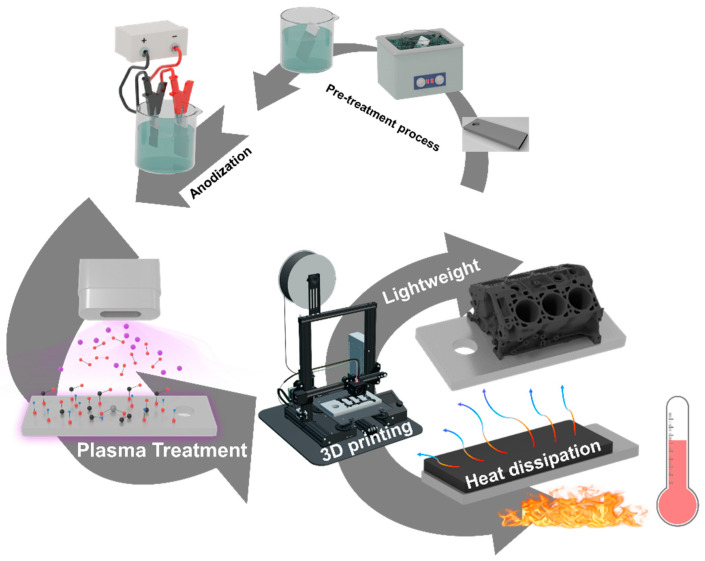
Manufacturing process of metal–composite hybrid structures for lightweight and heat dissipation applications.

Research on metal–polymer hybrid structures, however, has predominantly concentrated on enhancing their mechanical properties, such as weight reduction and bonding strength. To extend the application of metal–polymer hybrid structures across various industrial sectors, it is essential to diversify the bonded polymers and integrate functional composite materials. In this study, we developed innovative metal–polymer hybrid structures by 3D printing a variety of polymers—including polylactic acid, thermoplastic polyurethane, acrylonitrile butadiene styrene, polypropylene, and thermoplastic polyester elastomer—and conductive composite materials onto anodized metal surfaces, all of which possess high industrial applicability. To accomplish this, we employed several metal surface treatments, including anodizing with mixed electrolytes, plasma pre-treatment, and etching. Additionally, by leveraging the combined strengths of the metal and composite materials, we performed the first analysis of the enhanced thermal conductivity properties of these metal–composite hybrid structures. Finally, we fabricated a range of highly specialized prototypes, such as automotive and robotic components, as well as military units, to demonstrate the industrial potential of the metal–composite hybrid structures (Figure 1).

## 2. Experimental Methods

### 2.1. Materials

The aluminum alloy used in this study was AL6061-T6 (t3.0, Sungshin Jungmil, Incheon, Republic of Korea), composed of 97.6% aluminum, 0.8% magnesium, 0.7% iron, 0.4% silicon, and 0.4% copper, in accordance with the ASTM B209 standard. The surface treatment of the aluminum alloy involved sequential immersion in ethanol (98%, Samchun Chemical, Seoul, Republic of Korea), C-4000 (KG Chemical, Ulsan, Republic of Korea), NaOH (98%, Samchun Chemical, Seoul, Republic of Korea), and H_2_SO_4_ (95%, Samchun Chemical, Seoul, Republic of Korea). For the anodization process, a H_3_PO_4_ (85%, Samchun Chemical, Seoul, Republic of Korea) solution was used as the electrolyte, with H_2_O_2_ (99%, Samchun Chemical, Seoul, Republic of Korea) as an additive. Platinum (Premion, 0.1 mm, 99.99%) served as the counter electrode, and the aluminum alloy functioned as the working electrode. The polymer filament materials used for adhesion included polylactic acid (PLA, Cubicon, Seongnam, Republic of Korea), polypropylene (PP, Cubicon, Seongnam, Republic of Korea), thermoplastic polyurethane (TPU, Cubicon, Seongnam, Republic of Korea), acrylonitrile butadiene styrene (ABS, Cubicon, Seongnam, Republic of Korea), thermoplastic polyether-ester elastomer (TPEE-TRIEL, Samyang, Daejeon, Republic of Korea), and carbon-fiber-reinforced polylactic acid (CF-PLA, 3DXTECH, MALMÖ, Sweden).

### 2.2. Surface Treatment of Aluminum Alloys

The pre-treatment of aluminum specimens for surface modification was conducted following the protocols established in our previously published studies [20].

Following pre-treatment, the aluminum specimens underwent anodization in a H_3_PO_4_ solution. This was achieved by applying a constant current of 80 V and 0.1 A using an EA-PS 2384-05 B power supply (KMI system, Seongnam, Republic of Korea), with the pre-treated aluminum serving as the working electrode and a platinum plate as the counter electrode. The anodization was maintained for 15 min. Subsequently, the specimens were rinsed with deionized water and dried in an oven at 80 °C for 6 h. To control the pore size, 250 mM of H_2_O_2_ was incorporated into the anodization process, following the phosphoric acid protocol, and this was followed by identical rinsing and drying procedures. Surface activation was accomplished through plasma treatment using an ArP Series plasma generation device (APP, Hwaseong, Republic of Korea), which included an RF power generator and a plasma head unit to produce a plasma beam. The power of the plasma beam was 100 W and the plasma exposure time was standardized to 10 min. The plasma treatment was conducted in an O_2_/Ar atmosphere at a pressure of 50 Pa, with the specimen positioned 1.0 cm from the plasma source. Following the anodization and plasma treatment, the aluminum specimens underwent post-treatment with the 5 wt% NaOH solution for 10 min to further modify the surface characteristics. After NaOH etching, the specimens were thoroughly washed with deionized water and dried at 80 °C.

### 2.3. Preparation of Metal–Polymer Hybrid Structures

After completing the surface treatment of the aluminum samples, the polymer was precisely deposited onto the treated surfaces using a Core 200 3D printer (Making Tool, New Delhi, India). This process produced a structurally robust single-lap-joint configuration with a bonding area of 5.0 × 10.0 mm. For additional details on the metal–polymer hybrid fabrication process and shear strength measurements, please refer to Table 1, which categorizes the comprehensive information by factors and corresponding levels [21,22].

### 2.4. Bonding Strength Measurement

In compliance with the international standard ISO 19095-2:2015 [23], test specimens were fabricated according to the overlapped specimen guidelines, specifically designed as Type B. Shear strength measurements were performed using a precision shear testing machine (AGS-X series, Shimadzu, Kyoto, Japan) at a controlled rate of 5 MPa/min. To ensure accuracy, the final shear strength values were calculated as the average of the eight test results, with the highest and lowest values excluded to minimize the influence of outliers. The error bars in the figures represent the standard deviation from the mean data. All measurements were conducted under identical experimental conditions.

### 2.5. Heat Dissipation Characterization of Metal–Composite Hybrids

To evaluate the heat dissipation characteristics of the metal–polymer hybrids, two types of polymer filaments, PLA and CF-PLA, were 3D-printed onto an aluminum surface to form hybrid structures. The aluminum part used for the experiment had a rectangular shape with dimensions of 40.0 × 13.0 × 3.0 mm, and it was partially covered with a 30.0 × 13.0 × 3.0 mm CF-PLA layer. The rectangular shape was selected to fully cover the aluminum surface and to ensure consistent thermal dissipation comparisons.

During the experiment, the hybrid structure was heated to 60 °C to simulate the average operating temperature of the electronic devices. It was then allowed to cool naturally to a target temperature of 30 °C. The cooling rate was calculated as the reciprocal of the time taken to reach the target temperature. Temperature measurements were recorded at one-minute intervals using an infrared thermal imaging camera (FLIR ETS320, Teledyne Technologies, Thousand Oaks, CA, USA). All measurements were conducted in an open environment to reflect ambient cooling conditions.

### 2.6. Demonstration of Metal–Composite Hybrid Applications

To demonstrate the applications of the experiment, CF-PLA composite filament was employed to fabricate automotive, military, and robotic arm components via 3D printing techniques, resulting in the construction of metal–composite hybrid structures. The initial designs were sourced from Thingiverse (https://www.thingiverse.com). Detailed modifications necessary for effective bonding were conducted using the 3D modeling software, specifically AutoCAD (AutoCAD 2022) and Autodesk Fusion 360 (2.0.15290, c).

### 2.7. Characterization

A field-emission scanning electron microscope (FE-SEM, LEO SUPRA 55; GENESIS 2000, Carl Zeiss, EDAX, Jena, Germany) was utilized to examine the surface morphology before and after anodization and plasma surface treatment. Thermogravimetric analysis (TGA) was conducted to determine the carbon content in the composite filament. For the analysis, a 20 mg sample was loaded into a ceramic crucible. The temperature increased up to 600 °C at a constant heating rate of 5 °C/min under a nitrogen atmosphere. A gas flow rate of 50 mL/min was employed to maintain an inert environment and prevent oxidative degradation.

## 3. Results and Discussion

### 3.1. Surface Characterization of Anodized and Post-Treated Aluminum

**Figure 2:**
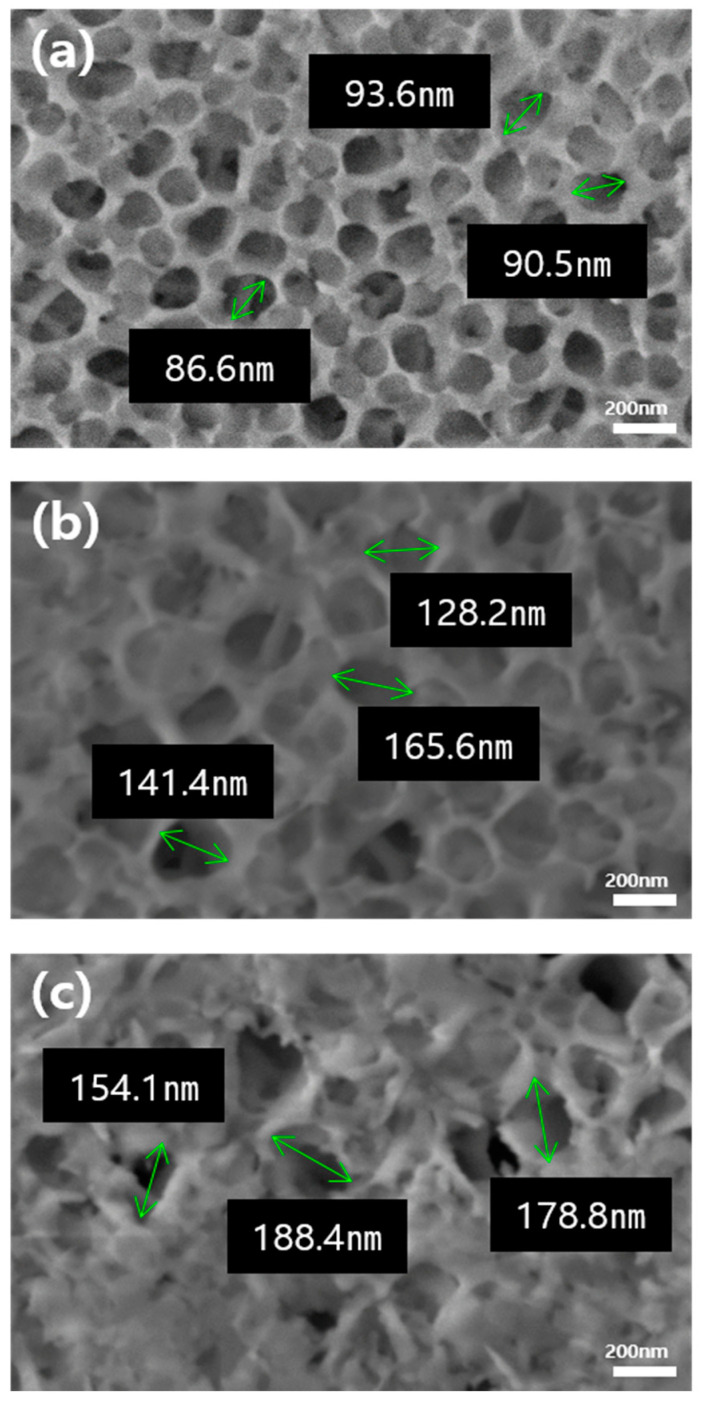
FE-SEM images of aluminum surface: (**a**) anodization in H_3_PO_4_; (**b**) anodization in a mixed electrolyte of H_3_PO_4_ and H_2_O_2_; and (**c**) anodization in H_3_PO_4_ and H_2_O_2_, followed by etching in NaOH.

Figure 2 presents the experimental results aimed at increasing pore size to enhance the penetration capacity of the injected polymer fluid, as observed through the field-emission scanning electron microscopy (FE-SEM) images. This modification was achieved either through additional surface treatment with NaOH or by introducing H_2_O_2_ into the phosphoric acid solution during the anodization process. In Figure 2a, the aluminum surface anodized solely with phosphoric acid exhibits pores with an average diameter of approximately 90 nm. Figure 2b shows the surface after anodization with the addition of 250 mM of H_2_O_2_ to the phosphoric acid solution, resulting in a significant increase in pore size, while the branch thickness remains largely unchanged. The average pore diameter in this case is about 145 nm. Finally, Figure 2c depicts the surface of the aluminum anodized in a solution of hydrogen peroxide and phosphoric acid, followed by immersion in NaOH for etching. This process led to an expanded average pore diameter of approximately 175 nm. However, the etching process also resulted in the formation of impurities within the pores.

In addition to controlling the atomic composition of the aluminum surface, regulating the size of the nanopores can influence the flow of polymer fluid across the surface, thereby enhancing the strength of the resulting bonds between aluminum and the molded polymer. Although the size of the nanopores can be regulated by adjusting the anodization parameters, such as time, voltage, and electrolyte concentration, the extent of this size variation is limited. Additionally, the improper control of these conditions may result in pore collapse [24]. Thus, in this study, an additive (H_2_O_2_) was introduced during the anodization process, followed by an etching treatment to increase the nanopore size on the aluminum surface. The FE-SEM images confirmed that hydrogen peroxide acted as an oxidizing agent, promoting the dissolution reaction and thereby increasing the nanopore size without causing pore collapse (Figure 2b). The formation of pores and the increase in pore size caused by the addition of H_2_O_2_ were investigated based on the anodizing mechanism [25].

H_3_PO_4_ ⇆ H_2_PO_4_^−^ + H^+^ (1)

Anode: 2H_2_O (l) → O_2_ (g) + 4H^+^ (aq) + 4e^−^ (2)

Cathode: 2H^+^ (aq) + 2e^−^ → H_2_ (g) (3)

Subsequently, the growth of the oxide layer was described as

Al + 3H^+^ → Al^3+^ (4)

Al^3+^ (aq) + 3OH^−^ (aq) → Al(OH)_3_ (s) (5)

2Al(OH)_3_ (s) → Al_2_O_3_ (s) + 3H_2_O (l) (6)

The dissolution of the oxide layer was described as

Al_2_O_3_ (s) + 6H^+^ (aq) → 2Al^3+^ (aq) + 3H_2_O (l) (7)

The addition of hydrogen peroxide led to the increase in the hydrogen ion concentration.

H_2_O_2_ (l) → O_2_ (g) + 2H^+^ (aq) + 2e^−^

On the other hand, etching with NaOH after the anodization process resulted in significant pore collapse and the formation of impurities (Figure 2c). Therefore, in this study, anodization with the addition of H_2_O_2_ was selected over NaOH post-treatment etching to enlarge the nanopore size for applications in metal bonding with composite materials, rather than for use with pure polymers.

### 3.2. Bonding Strength Measurements of Metal–Polymer Hybrids

**Figure 3:**
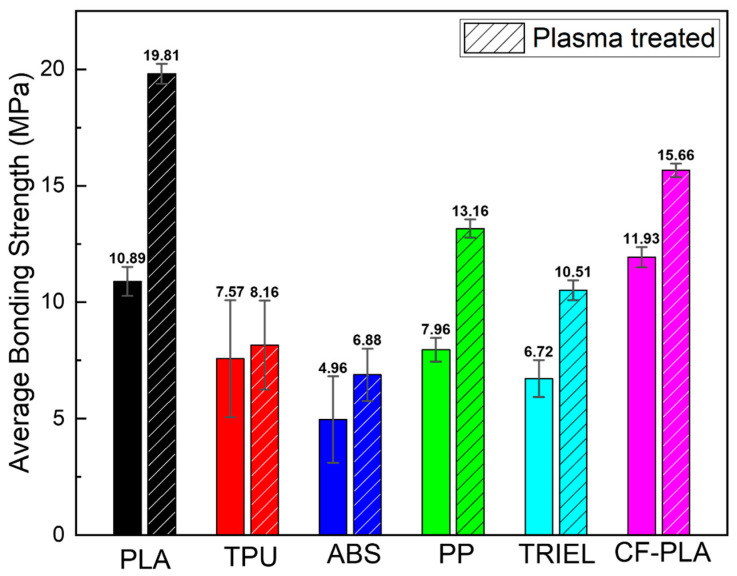
Comparison of the average bonding strengths for various types of polymer filament–aluminum hybrid structures. The bar with the dashed line indicates the bonding strengths for the hybrid structures with plasma-treated aluminum.

**Figure 4:**
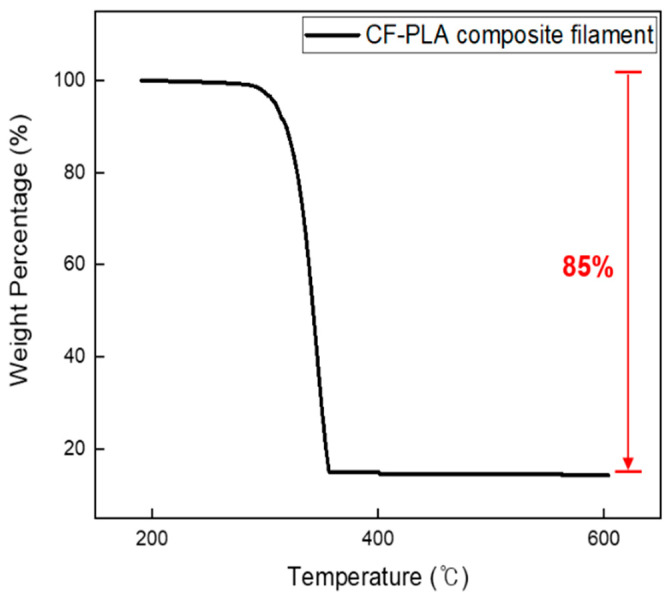
TGA of CF-PLA composite filament.

Surface-treated aluminum–polymer joint samples with a contact area of 5.0 × 10.0 mm^2^ were fabricated using 3D printing, in accordance with the ISO 19095-2:2015 international standards. Figure 3 presents the bonding strength results for various polymer filaments adhered to anodized aluminum, along with the effects of plasma treatment on each sample. The experiments utilized five commercially available filaments—PLA, TPU, ABS, PP, and TPEE—along with a carbon-fiber-infused PLA filament containing 15% carbon fiber, as confirmed by TGA in Figure 4 and Figure S1. When CF-PLA was bonded to anodized Al, it exhibited the highest bonding strength at 11.9 MPa, followed by PLA at 10.89 MPa. Following plasma treatment, the bonding strengths increased for all filaments, with PLA exhibiting the highest strength at 19.81 MPa. The bonding strength and increase rates are presented in Table 2. Notably, the hydrophobic properties of the carbon in the CF-PLA filament resulted in a relatively low increase rate of approximately 27% after plasma treatment. Conversely, the PLA filament, which initially exhibited a lower bonding strength than CF-PLA, demonstrated the most significant improvement following plasma treatment, with an increase of approximately 82% and a final bonding strength of 19.81 MPa.

The relatively low bonding strengths observed in ABS and TPEE can be attributed to their higher melting points (T_m_), which resulted in the premature termination of the bonding process before achieving the full penetration of polymer filaments into the porous Al layer. Consequently, despite the successful fabrication of the metal–polymer hybrid structures, the interaction between the polymer filaments and the porous layer was compromised by the premature solidification of the materials, resulting in diminished bonding strengths. This phenomenon underscores the critical role of material-specific processing parameters in achieving optimal bonding outcomes, particularly when the melting points of the materials approach or exceed the maximum temperature limit of the 3D printer nozzle.

**Figure 5:**
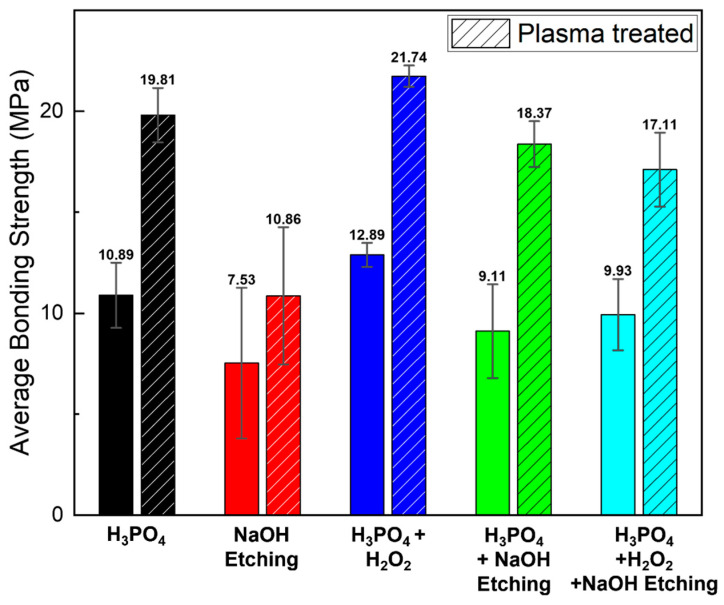
Comparison of the average bonding strengths of PLA–aluminum hybrid structures across different anodization electrolytes and surface treatments. The bonding strength of the plasma-treated structure is indicated by a dashed line.

Figure 5 and Figure S2 present the bonding strength results of the hybrid structure samples, where the PLA polymer was bonded to aluminum metals prepared through various surface treatment methods, including H_3_PO_4_ anodization, NaOH etching, H_3_PO_4_/H_2_O_2_ anodization, and etching followed by anodization. To verify the effect of plasma treatment on various hybrid structure samples, the bonding strength data were presented separately before and after plasma treatment. Anodization with H_3_PO_4_ alone resulted in a bonding strength of 10.81 MPa. After plasma treatment, the bonding strength increased significantly to 19.81 MPa. The aluminum and PLA bonds subjected to NaOH etching exhibited weak bonding strengths both before and after plasma treatment. The hybrid structure samples subjected to NaOH etching exhibited lower bonding strengths both before (7.53 MPa) and after plasma treatment (10.86 MPa), attributed to the absence of nanopores. On the other hand, introducing H_2_O_2_ to H_3_PO_4_ during anodization increased the bonding strength to 12.89 MPa. Following plasma treatment, the bonding strength further improved to 21.74 MPa, surpassing the values observed for samples anodized solely with H_3_PO_4_.

Furthermore, following NaOH etching and subsequent H_3_PO_4_ anodization, the bonding strength was measured to be 9.11 MPa. The application of the plasma treatment resulted in a more than twofold increase in bonding strength, reaching 18.37 MPa. Lastly, when anodization was conducted in a mixture of H_3_PO_4_ and H_2_O_2_, followed by etching, the bonding strength reached 9.93 MPa. After subsequent plasma treatment, the bonding strength further increased to 17.11 MPa.

The comparison of the average bonding strengths of the PLA–aluminum hybrid structure samples shown in Figure 5 with the FE-SEM images of aluminum surfaces in Figure 2 allowed for an analysis of how aluminum surface treatment influenced the bonding strengths of hybrid structure samples. The black bar graph in Figure 5 shows the results from a PLA–aluminum hybrid structure anodized with H_3_PO_4_, while the blue bar graphs were derived from anodization using a mixture of H_3_PO_4_ and H_2_O_2_. Adding H_2_O_2_ to the H_3_PO_4_ solution increased the average pore size from 90 nm to 145 nm, resulting in an enhancement of bonding strength from 10.81 MPa to 12.89 MPa. Similarly, plasma surface treatment increased the binding strength from 19.81 MPa to 21.74 MPa under the same anodizing conditions, suggesting that the enlarged pore diameter allowed for deeper penetration of the injected polymer fluid into the anodized film, thereby enhancing adhesion. In contrast, when anodized aluminum samples treated with H_3_PO_4_ and H_2_O_2_ were subsequently etched in NaOH, the average pore diameter increased to 175 nm, as shown in Figure 2c. However, the bonding strength indicated by the cyan bar graph in Figure 5 did not exceed 10.0 MPa due to surface impurities, whereas the bonding strength achieved after plasma treatment was 17.11 MPa. Data analysis concluded that the anodization process, particularly when using a solution with added H_2_O_2_ in addition to H_3_PO_4_, significantly enhanced the bonding strength. The increase in pore diameter resulting from this specific anodization condition indicated improved adhesion properties, emphasizing the importance of tailoring anodization parameters to prepare robust hybrid structure products.

### 3.3. Heat Dissipation Applications of Metal–Polymer Hybrid Structures

The heat dissipation experiment conducted a comparative analysis of the cooling rates among various reference materials and metal–polymer hybrids produced under different conditions. The reference materials used included bare aluminum, commercially available thermal interface material (TIM), PLA, and CF-PLA hybrids. The thermal conductivity values for each material are summarized in Table 3. TIM, a commonly used adhesive for efficient heat dissipation in central processing units (CPUs), exhibited a thermal conductivity of 12.8 W/mK [26]. PLA, characterized as an insulating polymer, displayed a thermal conductivity of 0.193 W/mK. In this study, a composite material, CF-PLA [27,28,29], was prepared by incorporating carbon fibers (15 wt%) with a thermal conductivity of 100 W/mK or higher. The CF-PLA composite demonstrated enhanced thermal conductivity relative to PLA, reaching a value of 0.225 W/mK.

**Figure 6:**
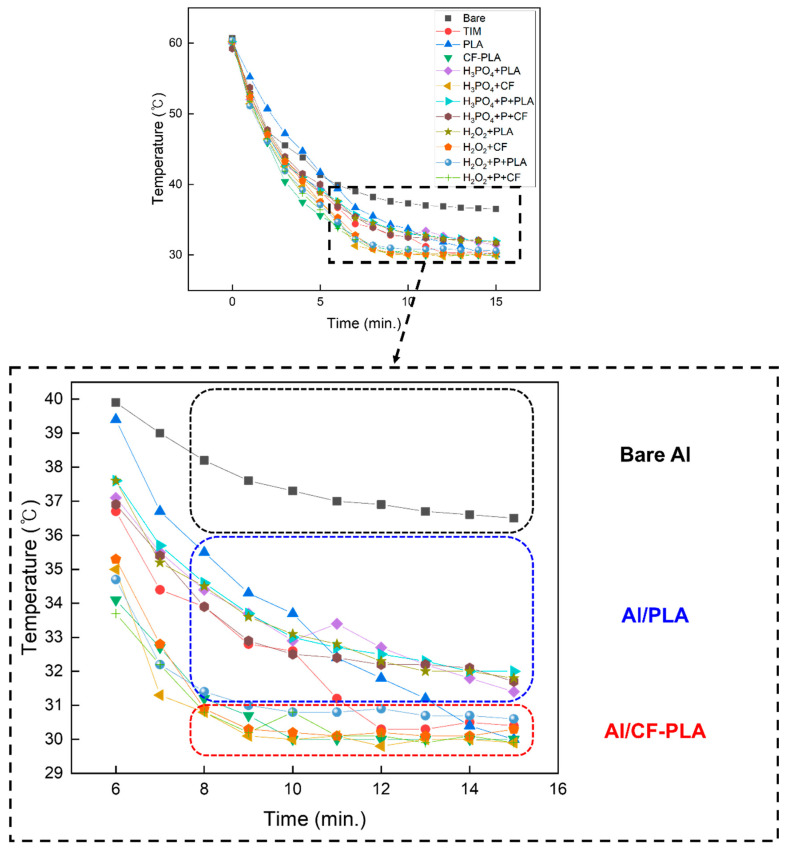
Temperature profile of bare Al, metal–polymer, and metal–composite hybrid structures recorded per minute, with variations in anodizing electrolytes, plasma treatment, and polymer injection.

The findings from the heat dissipation experiment are illustrated in Figure 6, which displays the results over a 15 min period, along with an enlarged line graph focusing on the temperature saturation region. The analysis of the data in these figures revealed that bare aluminum exhibited a gradual cooling process, ultimately stabilizing at 36 °C after approximately 15 min. In contrast, the hybrid structures formed by bonding PLA onto aluminum, represented by the blue square in Figure 6, demonstrated an enhanced heat dissipation performance. The PLA-associated adhesives reached a stabilized temperature at 32 °C after a cooling period exceeding 10 min, showcasing a marked improvement in heat dissipation compared to the bare aluminum. Finally, the most noteworthy observation pertained to the hybrid structure of Al/CF-PLA, highlighted in the red square. This metal–composite hybrid exhibited the highest cooling rate, reaching a reduced temperature of 30 °C and achieving temperature stabilization within 8 min. These findings emphasized the superior heat dissipation capability of the CF-PLA conjugate, indicating its potential as an effective solution for applications that demand rapid and efficient cooling.

**Figure 7:**
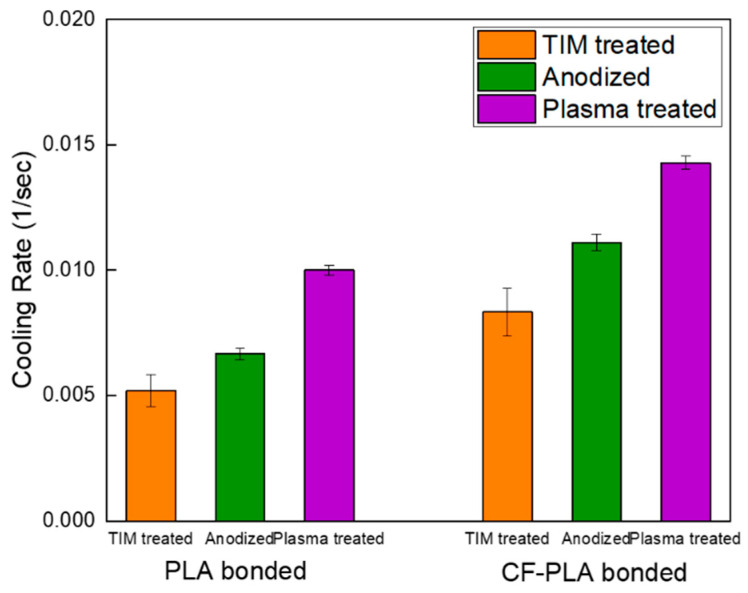
Heat dissipation rate of metal–polymer and metal–composite hybrid structures: Al/PLA (left) and Al/CF-PLA (right), prepared using TIM treatment, anodization, and plasma treatment.

Figure 7 compares the heat dissipation rates of metal–polymer and metal–composite hybrid samples prepared by anodization to those treated with TIM adhesive with a mixed solution of phosphoric acid and hydrogen peroxide. This study also evaluated the heat dissipation performance of anodized surface treatments in comparison to plasma surface treatments.

When comparing samples bonded using TIM as reference points, the anodized and plasma-treated samples exhibited superior heat dissipation properties [30]. These findings underscore the critical role of the hybrid structure formed between the porous metal surfaced and the injected polymer through anodization in achieving an enhanced thermal conductive performance. Additionally, bonding with CF-PLA filaments, rather than standard PLA filaments, demonstrated superior heat dissipation. This improvement was attributed to the carbon fiber composite structure, which augmented the thermal and mechanical properties of the bonded polymers. Specifically, the heat dissipation rate of the metal–composite hybrid sample bonded with CF-PLA after plasma treatment was more than twice that of the Al-PLA sample bonded using TIM. Plasma treatment enhanced the fluidity of the polymer and composite materials by introducing numerous functional groups through surface activation, enabling deeper penetration. Consequently, this approach demonstrated excellent heat dissipation by reducing air layer thickness and achieving strong adhesion.

In summary, the enhanced heat dissipation characteristics of the metal–composite hybrid structure were achieved through the combined effects of anodization and plasma treatments of the metal surface, as well as the utilization of carbon-fiber-reinforced polymers.

## 4. Conclusions

In conclusion, this study effectively utilized mixed electrolyte anodization and plasma surface treatments to enhance the bonding strength and thermal dissipation properties of metal–composite hybrid structures. The introduction of H_2_O_2_ during the anodization process significantly increased the nanopore size on the aluminum surfaces, enhancing the bonding strength between aluminum and polymer. Notably, the bonding strength increased from 10.81 MPa to 12.89 MPa by adding H_2_O_2_ to the H_3_PO_4_ anodization solution. Plasma treatment further enhanced the bonding strength to 21.74 MPa, demonstrating a substantial improvement due to the increased pore size allowing deeper polymer penetration. In addition, the integration of carbon-fiber-reinforced polymers within these structures offered significant advantages in applications requiring lightweight materials with excellent thermal management capabilities. The Al/CF-PLA hybrid structure achieved a higher cooling rate, stabilizing at 30 °C within 8 min, compared to other materials. This is particularly beneficial in sectors such as automotives, electronics, and military, where enhanced heat dissipation and structural integrity are crucial (Figure S3). These results underscore the potential of tailored anodization and surface treatment processes in developing robust and efficient metal–composite hybrid structures suitable for diverse industrial applications [31,32,33,34].

## Figures and Tables

**Table 1:** Factors and levels employed in the fabrication of metal–polymer hybrid structures.

Factor		Level					
3D Printing	Type of polymer filament	PLA	TPU	PP	ABS	TPEE	CF-PLA
	Nozzle temperature (°C)	210	200	220	260	260	200
	Bed temperature (°C)	100					
	Chamber temperature (°C)	60					
	Printing speed (mm/min)	5000					
Adhesion method/Area (m^2^)		Single lap joint/5.0 × 10.0					
Measuring speed (MPa/min)		5					

**Table 2:** Average bonding strengths of various polymer–metal hybrid structures and increase rates before and after the plasma treatment of aluminum.

	PLA		TPU		ABS		PP		TRIEL		CF-PLA	
Plasma treatment	X	O	X	O	X	O	X	O	X	O	X	O
Bonding strength (MPa)	10.89	19.81	7.57	8.16	4.96	6.88	7.96	13.01	6.98	10.50	11.90	15.16
Increase rate (%)	81.91		7.79		38.71		63.44		50.43		27.39	

**Table 3:** Thermal conductivity of reference materials.

Material	Thermal Conductivity (W/mK)
Thermal interface materials (TIM)	12.8
PLA	0.193
Carbon fiber	100~
CF-PLA	0.225

## Data Availability

The original contributions presented in this study are included in the article/Supplementary Materials. Further inquiries can be directed to the corresponding author.

## Supplementary Materials

The following supporting information can be downloaded at: https://www.mdpi.com/article/10.3390/mi16040399/s1, Figure S1. TGA analyses of PLA filament and carbon fiber. Figure S2. Raw force–displacement curves of PLA-aluminum hybrid structures across different anodization electrolytes and surface treatments, (a) before and (b) after plasma treatment. Figure S3. Applications of Al/CF-PLA hybrids in the fabrication of lightweight metal-composite hybrid structures: (a) and (b) military armor components, (c) and (d) automotive parts, and (e) engineering machinery components.
