# Supplementary material for: Metal Surface Treatments for Enhanced Heat Transfer in Metal–Composite Hybrid Structures
Source: Micromachines (Basel). 2025 Mar 29;16(4):399. doi: 10.3390/mi16040399 (PMC12029697; doi:10.3390/mi16040399)
Supplement: Supplementary file 1 [file micromachines-16-00399-s001.zip › micromachines-3527585-supplementary.pdf]

Supplementary data

Metal Surface Treatments for Enhanced Heat Transfer in Metal-Composite Hybrid Structures

Dong Hyun Kim[a],†, Wonhwa Lee[a],†, Jung Bin Park[a], and Jea Uk Lee[a,*]

[a] *Department of Advanced Materials Engineering for Information and Electronics, Integrated EducationInstitute for Frontier Science and Technology (BK21 Four), Kyung Hee University, Gyeonggi-do, 17104, Republic of Korea*

* Corresponding author: Jea Uk Lee; E-mail: leeju@khu.ac.kr
† These authors contributed equally to this work.

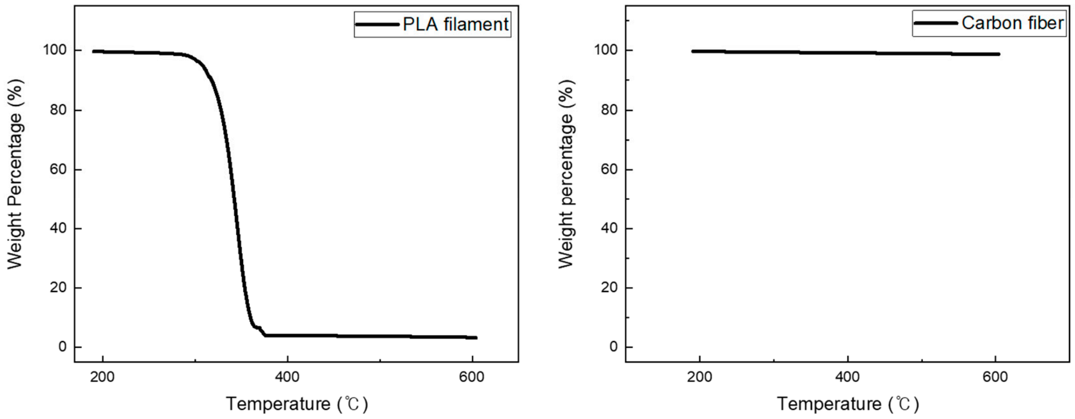

Figure S1. TGA analyses of PLA filament and carbon fiber.

To verify the decomposition characteristics of the composite filaments, thermogravimetric analysis (TGA) was conducted. As illustrated in Figure Sa, pure PLA was almost entirely decomposed at 600 °C, indicating complete thermal degradation. In contrast, pure carbon fiber exhibited minimal weight loss, retaining about 99.6% of its mass at 600 °C, which confirms its thermal stability. The CF-PLA composite filament showed a residual mass of approximately 15% at 600 °C, aligning well with the expected 15 wt% carbon fiber content. These results confirm that the PLA matrix is fully removed at 600 °C, while the carbon fiber remains intact, thereby validating the composition of the CF-PLA composite.

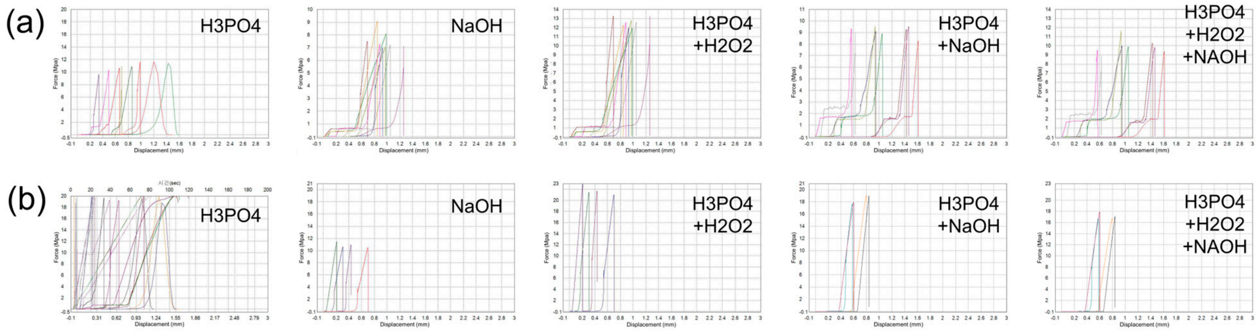

Figure S2. Raw force–displacement curves of PLA-aluminum hybrid structures across different anodization electrolytes and surface treatments, (a) before and (b) after plasma treatment.

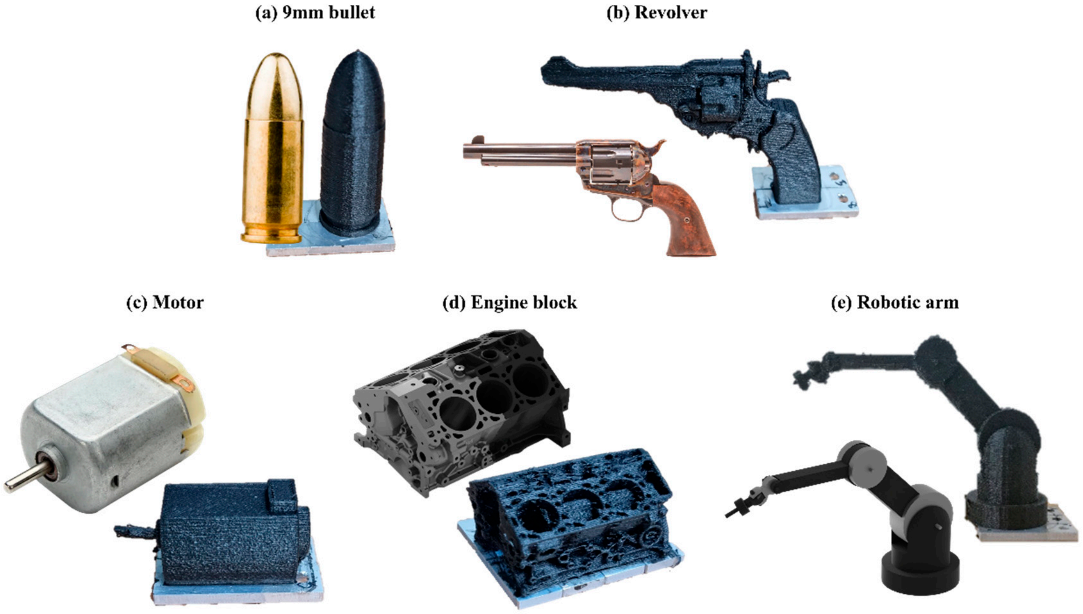

Figure S3. Applications of Al/CF-PLA hybrids in the fabrication of lightweight metal-composite hybrid structures: (a) and (b) military armor components, (c) and (d) automotive parts, and (e) engineering machinery components.

Lightweight composites application of metal-composite hybrid structures

The research explores the composites application of metal-composite hybrids, particularly those incorporating carbon fiber, as a strategy to reduce the weight of metallic materials. Figure S3

illustrates a range of products from diverse fields that utilize aluminum alloys, highlighting the potential applications of the developed technology.

Figure S3 (a) and Figure S3 (b) illustrate the integration of a 9.0 mm bullet and a revolver component into Al/CF-PLA hybrids, demonstrating potential applications within military contexts. Replacing bullet components, excluding the warhead, provides benefits such as extended range and reduced weight relative to conventional aluminum bullets. For firearms, including revolvers and rifles, this metal-composite hybrid technology facilitates weight reduction by enabling the production of specific components—such as gun barrels and muzzles, which are exposed to high temperatures during firing—using CF-PLA and Al treated with anodization. These advancements contribute to enhanced mobility and overall operational performance for military personnel.

Figure S3 (c) and Figure S3(d) demonstrate the incorporation of Al/CF-PLA hybrids for the replacement of specific motor and engine block components, highlighting its potential applications in the automotive and industrial device sectors. Notably, certain automotive manufacturers have made strides toward commercializing components such as brakes and wheels using polymer composites reinforced with carbon fibers. This approach contributes to enhanced fuel efficiency and overall vehicle performance. The application of this technology enables the production of lighter vehicle bodies by substituting smaller, complex parts with carbon composite structures.

In Figure S3(e), the integration of a robotic arm onto an anodized substrate demonstrates its potential application in engineering fields. A cost-effective advantage can be realized if the robotic arm—a critical component in the manufacturing industry—incorporates carbon composite materials in all sections except those required to handle heavy loads. This substitution has the potential to reduce manufacturing costs while maintaining structural integrity in high-stress areas.
